# Vitamin C Sensitizes Pancreatic Cancer Cells to Erastin-Induced Ferroptosis by Activating the AMPK/Nrf2/HMOX1 Pathway

**DOI:** 10.1155/2022/5361241

**Published:** 2022-07-19

**Authors:** Yawen Liu, Pu Huang, Zheng Li, Chunhui Xu, Huizhi Wang, Baoqing Jia, Aihua Gong, Min Xu

**Affiliations:** ^1^Department of Gastroenterology, Affiliated Hospital of Jiangsu University, Jiangsu University, Zhenjiang, China 212001; ^2^Medical School of Chinese PLA, Beijing, China 100853; ^3^Department of General Surgery, The First Medical Centre, Chinese PLA General Hospital, Beijing, China 100853; ^4^Department of Cell Biology, School of Medicine, Jiangsu University, Zhenjiang, China 212013

## Abstract

Ferroptosis is a type of regulated cell death that displays a promising therapeutic pathway for drug-resistant tumor cells. However, some pancreatic cancer (PC) cells are less sensitive to erastin-induced ferroptosis, and normal pancreatic cells are susceptible to this newly discovered cell death. Therefore, there is an urgent need to find drugs to enhance the sensitivity of these PC cells to erastin while limiting side effects. Here, we found that the oxidized form of vitamin C-dehydroascorbic acid (DHA) can be transported into PC cells expressing high levels of GLUT1, resulting in ferroptosis. Moreover, pharmacological vitamin C combined with erastin can synergistically induce ferroptosis of PC cells involving glutathione (GSH) reduction and ferrous iron accumulation while inhibiting the cytotoxicity of normal cells. Mechanistically, as a direct system Xc^−^ inhibitor, erastin can directly suppress the synthesis of GSH, and the recycling of vitamin C and DHA is performed through GSH consumption, which is denoted as the classical mode. Furthermore, oxidative stress induced by erastin and vitamin C could enhance the expression of HMOX1 via the AMP-activated protein kinase (AMPK)/nuclear factor erythroid 2-related factor 2 (NRF2) pathway to increase the labile iron level, which is named the nonclassical mode. In vivo experiments showed that erastin and vitamin C can significantly slow tumor growth in PC xenografts. In summary, the combination of erastin and vitamin C exerts a synergistic effect of classical and nonclassical modes to induce ferroptosis in PC cells, which may provide a promising therapeutic strategy for PC.

## 1. Introduction

Pancreatic cancer (PC) is a fatal and common disease of the digestive system with a 5-year survival rate of less than 10% [1]. The lack of effective early detection methods makes more than 80% of patients with pancreatic cancer often unsuitable for surgical resection [2]. Chemotherapy is used as the primary treatment for advanced pancreatic cancer; however, the emergence of chemotherapy resistance and side effects limits the efficacy of chemotherapy, leaving patient survival rates far from satisfactory [3, 4]. Therefore, it is imperative to explore alternative combination treatment strategies to improve outcomes.

Ferroptosis is a newly discovered cell death process that features iron-dependent, intracellular accumulation of lipid reactive oxygen species (ROS) [5]. It involves a unique combination of morphological, biochemical, and genetic features that differ significantly from other cell death forms, such as apoptosis and necrosis [6]. The mechanism of ferroptosis is mainly related to the disruption of iron metabolism, imbalance of the amino acid antioxidant system, and lipid peroxide accumulation [7]. The cystine/glutamate antiporter (system Xc^−^) located in the cell membrane is involved in the synthesis of glutathione (GSH), and glutathione peroxidase 4 (GPX4) uses GSH as a substrate to reduce lipid peroxides to normal lipids, which prevents lipid peroxide accumulation and inhibits the occurrence of ferroptosis [8, 9]. Therefore, ferroptosis is caused by the insufficient ability of GPX4 to scavenge peroxides and/or excessive lipid peroxidation reactions, causing lipid peroxide accumulation and eventually inducing cell death [10, 11]. Moreover, ferroptosis can also be induced through the intracellular ferrous iron level in cancer cells, called the nonclassical ferroptosis induction mode. When disorders of iron metabolism cause an increase in intracellular free iron, iron catalyzes the production of ROS through the Fenton reaction, which further promotes lipid peroxidation, causing lipid peroxide accumulation and inducing ferroptosis [12].

Ferroptosis, as a promising therapeutic pathway for drug-resistant tumor cells, has attracted attention recently. As an efficient ferroptosis inducer, erastin can mediate ferroptosis, which inhibits system Xc^−^, blocks the voltage-dependent anion channel (VDAC), and activates p53 [13, 14]. Ferroptosis inhibits the proliferation of various malignant cancer cells, such as breast cancer, liver cancer, and lung cancer [15–17]. Nevertheless, a variety of erastin-insensitive cancer cells have also been reported in previous studies. In particular, our research found that the PC cell lines PaTu8988 and BxpC3 were insensitive to erastin-induced ferroptosis and that high-dose erastin could affect normal pancreatic cells [18]. Therefore, there is an urgent need to discover drugs to enhance the sensitivity of these cells to erastin. High-dose vitamin C (0.1-100 mM) has been demonstrated to enhance lipid peroxidation against cancer cells by GSH consumption and iron-mediated ROS generation [19]. Vitamin C can also effectively provide electrons to Fe^3+^ to regenerate Fe^2+^. This could trigger ferroptosis by increasing ferrous iron levels in colorectal cancer cells and promote the lethal metabolic cell death program induced by ATP depletion and oxidative stress when it is combined with cetuximab [20]. In addition, vitamin C could sensitize cancer cells to radiation therapy and chemotherapy and ameliorate normal tissue injury during tumor therapy [21, 22]. Therefore, we hypothesize that the strategy of combining classical and nonclassical models could increase the sensitivity of PC cells to ferroptosis while limiting side effects.

The present study showed that PaTu8988 and BxpC3 were insensitive to erastin-induced ferroptosis and that erastin exhibited cytotoxicity against normal pancreatic cells and MEFs. Specifically, we further demonstrated that vitamin C treatment synergistically with erastin not only promoted the sensitivity of PC cells to ferroptosis but also protected normal pancreatic cells and MEFs from erastin-induced ferroptosis. Additionally, we proved that combination treatment with vitamin C and erastin consumed intracellular GSH and led to ferrous iron accumulation by regulating the AMPK/NRF2/HMOX1 pathway to promote ferroptosis in PC cells. Our study develops a potential therapeutic strategy for PC patients via vitamin C- and erastin-induced ferroptosis.

## 2. Materials and Methods

### 2.1. Cell Lines and Cell Culture

The human pancreatic cancer cell lines PaTu8988, BxPC3, and PANC1; mouse embryonic fibroblasts (MEFs); and the mouse pancreatic cancer cell line Panc02 were obtained from Shanghai Institutes for Biological Sciences (CAS), and the immortalized pancreatic ductal epithelial cell line H6C7 was obtained from the American Type Culture Collection. All cells were tested and authenticated by short tandem repeat DNA typing analysis. BxPC3, H6C7, and Panc02 cells were cultured in RPMI-1640 medium (HyClone, Beijing, China); PANC1, PaTu8988, and MEF cells were cultured in DMEM (HyClone, Beijing, China). All media were supplemented with 10% fetal bovine serum (Gibco, Carlsbad, CA, USA), 100 units/ml penicillin, and 100 mg/ml streptomycin (Solarbio, Beijing, China). Cells were cultured in a humidified 5% CO_2_ incubator at 37°C.

### 2.2. Plasmid Construction

The GLUT1 shRNA 1-3 oligos produced from Shanghai (China) were first annealed into double strands and then cloned into the GV248-puro vector. The GLUT1 shRNA clones used in this study are described in Table S1.

### 2.3. Cell Counting Kit-8 (CCK-8) Proliferation Assay

Cells (3 × 10^3^) were seeded into a 96-well culture plate and treated with erastin (HY-15763, MedChemExpress) and/or vitamin C with or without deferoxamine (DFO; D9533, Sigma–Aldrich) for 24 h. To assess the efficiency of cell proliferation, Cell Counting Kit-8 (CCK8, A311-01/02, Vazyme) was used according to the manufacturer's protocol. The absorbance value (OD) was measured with a microplate reader at 450 nm.

### 2.4. Glutathione (GSH) Assay

We used a reduced glutathione (GSH) assay kit (A006-2-1, Nanjing Jiancheng Bioengineering Institute) to measure intracellular GSH levels. Cells (3 × 10^5^) were seeded into a 6-well plate with erastin and/or vitamin C for 24 h. The cells were harvested, and 0.3 ml PBS was added to the homogenization medium. Next, we took 100 *μ*l supernatant for determination and generated a standard curve of GSH concentration. The plate was incubated for 5 min after mixing the sample and reagents, and the absorbance value was measured at 405 nm. Then, the exact GSH concentration of different cell lines was calculated based on the GSH standard curve following the manufacturer's instructions.

### 2.5. Lipid Peroxidation Assay

BODIPY 581/591 C11 dye (Invitrogen, D3861) was used to assay the lipid peroxidation level of cells according to the manufacturer's instructions. Briefly, cells were cultured in a 6-well plate containing 2 *μ*M BODIPY 581/591 C11 dye, washed with PBS, and trypsinized after incubating for 30 minutes at 37°C. Then, the lipid peroxidation level was measured by flow cytometry as shown in the figures. According to the manufacturer's instructions, the MDA concentration in cell or tumor lysates was detected by a malondialdehyde (MDA) assay kit (A003-1-2, Nanjing Jiancheng Bioengineering Institute).

### 2.6. Iron Assay

For intracellular iron detection, an iron assay kit (MAK025-1KT, Sigma–Aldrich) and BioTracker 575 Red Fe^2+^ Dye (SCT030, Sigma–Aldrich) were used following the manufacturer's instructions.

### 2.7. RNA Sequencing

BxPC3 cells (3 × 10^5^) were seeded into a 6-well culture plate and treated with erastin and/or vitamin C for 48 h. Cells were washed with cold PBS twice, and total RNA was isolated using RNAiso Plus (Takara) following the manufacturer's protocol. After verifying its concentration and integrity, the qualified RNA samples were subjected to PCR amplification to construct a cDNA library. Cluster generation and sequencing were performed on a NovaSeq 6000 S4 platform using a NovaSeq 6000 S4 Reagent kit V1.5. To guarantee the data quality that was used for analysis, the useful PerlScript was used to filter the original data to remove low-quality sequences. The reference genomes and the annotation file were downloaded from the ENSEMBL database (http://www.ensembl.org/index.html).

### 2.8. qRT–PCR

Total RNA was isolated using RNAiso Plus (Takara) following the manufacturer's protocol. A RevertAid First-Strand cDNA Synthesis Kit (Thermo Fisher Scientific) was used for reverse transcription according to the manufacturer's recommendations. SYBR green-based real-time PCR was then performed in triplicate using SYBR Green Master Mix (Vazyme), and *β*-actin was used as the internal control. The primers for qRT–PCR are shown in Table S2.

### 2.9. Western Blotting

The Western blotting assay was performed as described previously [23]. The antibodies used were rabbit anti-GPX4 (Abcam, ab125066), rabbit anti-SLC7A11 (CST, 12691S), rabbit anti-*β*-tubulin (CST, 6181S), rabbit anti-CP (ABclonal, A20229), rabbit anti-HMOX1 (Abcam, ab172730), rabbit anti-NRF2 (Proteintech, 16396-1-AP), rabbit anti-NCOA4 (Abcam, ab86707), rabbit anti-phospho-AMPK*α* (Thr172) (CST, 2535S), rabbit anti-AMPK*α* (CST, 5831S), and rabbit anti-FTH1 (CST, 4393S).

### 2.10. Preparation of Nuclear and Cytoplasmic Protein Extracts

The nuclear and cytoplasmic protein extracts were prepared by a Nuclear Protein Extraction Kit (R0050, Solarbio, China) according to the manufacturer's instructions. The cytoplasmic extract and the nuclear extract were collected and stored at -80°C until use.

### 2.11. Immunofluorescence Analysis

For immunofluorescence, cells were plated on coverslips in 24-well plates for 48 h. After washing twice with PBS, cells were fixed in ice-cold 3% paraformaldehyde for 30 min at room temperature and then blocked with 3% BSA for 1 h. After rewarming for 1 h, the cells were incubated with specific secondary antibodies for 2 h at 37°C in the dark. Cells were stained with DAPI (1 *μ*g/ml, Pierce, IL, USA) for 5 min at room temperature after washing three times with PBS. Fluorescence images were taken with a confocal microscope (DeltaVision Elite, GE Healthcare).

### 2.12. Xenograft Tumor Models

All animal experiments were approved by the Ethics Committee of Jiangsu University. To investigate the role of the combination of erastin and vitamin C in inducing ferroptosis, Panc02 cells (1 × 10^5^ cells/site) were transfected and subcutaneously injected into 4-week-old C57BL/6 mice to generate xenografts. When the tumors reached a volume of 50-100 mm^3^, the mice were randomly divided into four groups (five mice per group) and treated with DMSO (control), imidazole ketone erastin (IKE, MedChemExpress), vitamin C, or a combination of erastin and vitamin C. Mice were treated with 80 *μ*l (400 *μ*M) erastin by intratumoral injection and/or 4 g/kg vitamin C by intraperitoneal injection every 2 days. The tumor size was measured every 2 days after injection until the endpoint at day 14 and calculated with the following formula: length × (width^2^)/2. The hearts, lungs, spleens, kidneys, and liver were fixed with 4% paraformaldehyde for hematoxylin and eosin staining.

### 2.13. Hematoxylin–Eosin (H&E) Staining

Paraffin-embedded tissues were sectioned for hematoxylin–eosin (H&E) staining assays. The thickness of the sections was 5 *μ*M. The histopathological changes in the sections were viewed under an Olympus microscope (BX51).

### 2.14. Statistical Analysis

All data are presented as the mean ± s.d. from at least three independent experiments. Comparisons between groups were analyzed using Student's *t* test (two groups) or one-way ANOVA (multiple groups) using GraphPad Prism 5 software. *P* < 0.05 was considered statistically significant.

## 3. Results

### 3.1. Vitamin C Induces Cytotoxicity in PC Cells but Not in Normal Pancreatic Ductal Epithelial Cells or Mouse Embryonic Fibroblasts

**Figure 1:**
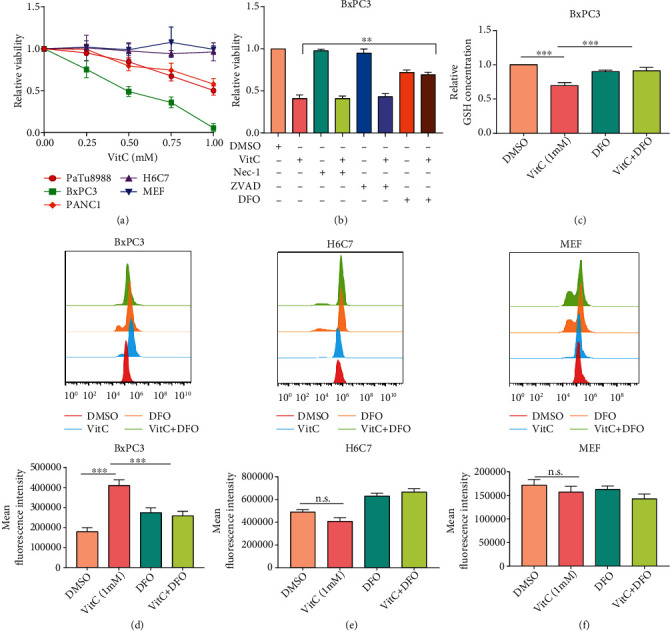
Vitamin C selectively induces ferroptosis in pancreatic cancer cells. (a) Cell viability was assessed by CCK-8 assay after treatment with various concentrations of vitamin C. (b) Cell viability was detected by CCK-8 assay after treatment with vitamin C with or without different cell death inhibitors. (c) GSH levels were measured in BxPC3 cells treated with vitamin C in the presence or absence of DFO. (d–f) Flow cytometry was performed to detect lipid ROS levels, and the quantification of fluorescence intensity is shown.

To study the sensitivity of erastin on the proliferation of different PC cell lines, we treated PC cell lines (PANC1, PaTu8988, and BxpC3) with different concentrations of erastin for 24 h and assessed cell viability via CCK-8 assay. The results showed that PANC1 cells were sensitive to erastin, while PaTu8988 and BxpC3 cells were insensitive to erastin-induced ferroptosis (Figure S1A). Considering that vitamin C exerts a high capacity in cancer therapy, we wondered whether vitamin C combined with erastin could increase the sensitivity of PC cells to ferroptosis. To explore the potential cytotoxicity of vitamin C against different cell lines, we first analyzed the viability of PaTu8988, BxPC3, PANC1, H6C7, and MEF cells via a CCK-8 assay. The results indicated that vitamin C induced cell death in a dose-dependent manner in PaTu8988, BxPC3, and PANC1 cells without affecting H6C7 and MEF cells (Figure 1(a)). To further determine the detailed mechanism of vitamin C-mediated cytotoxicity, various pharmacological inhibitors were used to identify the type of cell death, including ferroptosis, apoptosis, and necroptosis inhibitors (DFO, ZVAD, and Nec-1, respectively). Vitamin C-induced cell death was significantly restored by DFO but not by ZVAD or Nec-1 (Figures 1(b) and S1B). To explore ferroptosis induced by vitamin C, we measured the levels of GSH, which plays an essential role in repairing oxidative damage. As demonstrated in Figures 1(c) and S1C, vitamin C significantly decreased GSH levels in PaTu8988 and BxPC3 cells, while the iron chelator deferoxamine (DFO) treatment could rescue the decreased GSH level caused by vitamin C. Meanwhile, we checked the lipid ROS level by BODIPY 581/591 C11 fluoroprobe, and the results demonstrated that vitamin C increased the cellular lipid ROS production in PaTu8988 and BxPC3 cells but not in H6C7 and MEF cells (Figures 1(d)–1(f) and S1D). DFO effectively inhibited the ROS generation caused by vitamin C. The results further verified that vitamin C could induce ferroptosis in PC cells by producing excessive intracellular ROS.

### 3.2. Vitamin C Selectively Kills Pancreatic Cancer Cells via GLUT1

**Figure 2:**
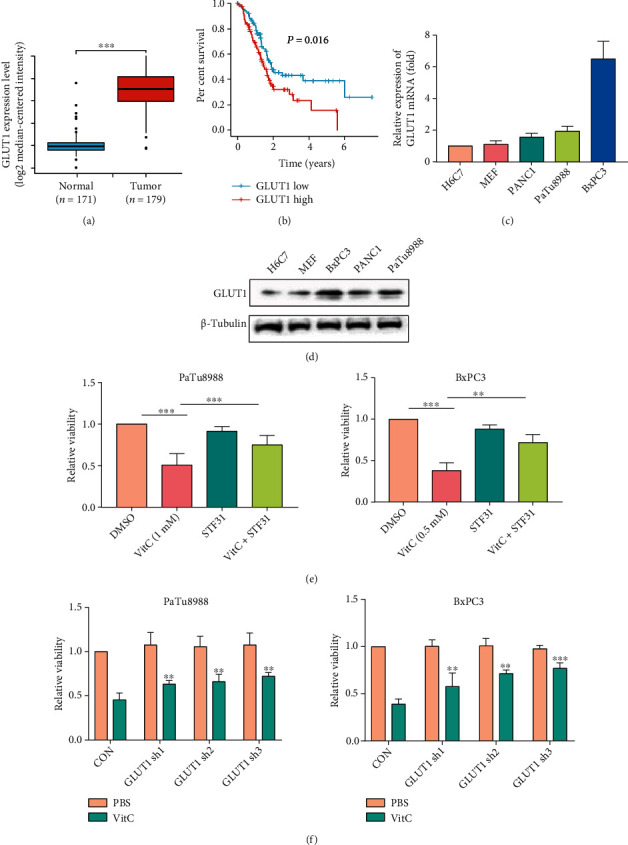
Vitamin C selectively kills pancreatic cancer cells via GLUT1. (a) TCGA database analysis showed that GLUT1 expression was higher in PC tumor tissues than in normal tissues. (b) The Kaplan–Meier survival curves indicated a significant correlation between high GLUT1 expression and low overall survival of PC patients (*P* = 0.016). (c, d) The relative expression of GLUT1 mRNA and protein in H6C7 cells, MEFs, and different PC cell lines. (e) Cell viability was detected by CCK-8 assay after treatment with vitamin C and/or STF-31. (f) Cell viability was detected in GLUT1-downregulated PaTu8988 and BxPC3 cells treated with vitamin C or not (^∗∗^*P* < 0.01, ^∗∗∗^*P* < 0.001).

A growing number of studies during the past decade have demonstrated that pharmacological concentrations of vitamin C are effective in killing cancer cells *in vitro* and slowing tumor growth *in vivo* [24, 25]. However, the mechanism by which cancer cells are sensitive to vitamin C while normal cells remain resistant is unclear and requires further study. Unless reducing agents are added, vitamin C is oxidized to dehydroascorbic acid (DHA) in cell culture media [26]. Unlike vitamin C, DHA is mainly transported via glucose-facilitated transporters (GLUTs), mainly GLUT1, into the cells, reducing to vitamin C at the expense of redox balance disruption and impaired cell viability [27, 28]. Overexpressing GLUT1 in KRAS and BRAF mutant cells reportedly results in massive DHA uptake, ultimately leading to ROS production and cancer cell death [29]. To explore the mechanism by which vitamin C selectively attacks PC cells, we first analyzed the expression of GLUT1 in PC tissues with The Cancer Genome Atlas (TCGA) database. We found that GLUT1 was highly expressed in PC tumor specimens compared with normal tissues, and it was a negative prognostic factor for PC overall survival (Figures 2(a) and 2(b)). We measured GLUT1 mRNA levels in H6C7 cells, MEFs, and three different PC cell lines by qPCR. The results indicated that GLUT1 levels were significantly higher in PC cell lines than in H6C7 and MEF cells (Figure 2(c)). Next, we tested the relative protein levels of GLUT1 in H6C7 cells, MEFs, and PC cell lines using Western blotting. As shown in Figure 2(d), the GLUT1 protein level was the highest in BxPC3 cells, followed by PaTu8988 and PANC-1 cells, and the lowest in H6C7 and MEFs cells (Figure 2(d)). STF31, a GLUT1-specific inhibitor, significantly alleviated the decline in cell death caused by vitamin C in PaTu8988 and BxPC3 cells (Figure 2(e)). In addition, we transfected sh-GLUT1 or sh-CON into PaTu8988 and BxPC3 cells to examine GLUT1 expression. Compared with the sh-CON group in PaTu8988 and BxPC3 cells, GLUT1 protein and mRNA levels were decreased in sh-GLUT1 cells (Figures S2A–D). The cell viability analysis indicated that vitamin C-induced cell death was inhibited in GLUT1-silenced PC cells (Figure 2(f)). GLUT1-OE or CON plasmids were transfected into H6C7 and PANC1 cells, and the GLUT1 protein levels significantly increased (Figures S2E and F). The cell viability analysis showed that GLUT1 overexpression enhanced the toxicity of vitamin C in H6C7 and PANC1 cells (Figures S2G and H). These findings might explain why vitamin C could selectively kill PC cells rather than normal pancreatic cells.

### 3.3. The Combination of Erastin and Vitamin C Inhibits Proliferation in PC Cells

**Figure 3:**
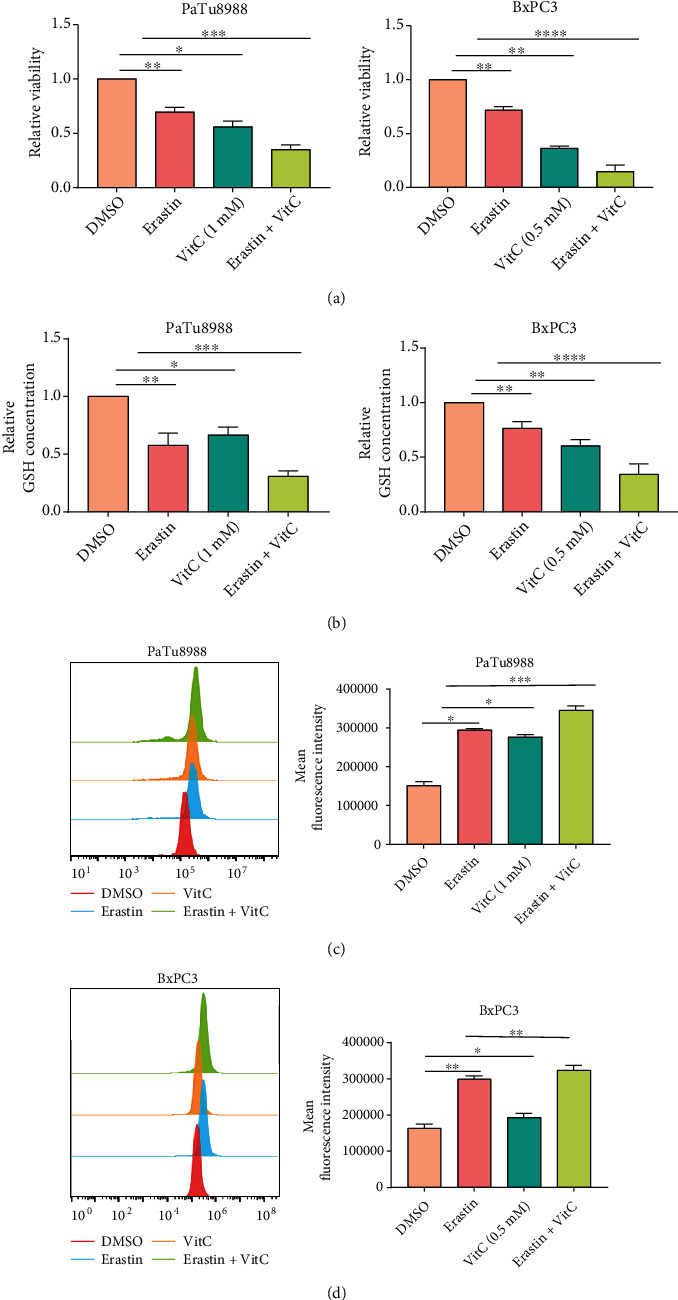
The synergistic effect after combination treatment with vitamin C and erastin. (a–d) PaTu8988 and BxPC3 cells were treated with a mono- or combination of erastin and vitamin C for 24 h. Cell viability (a), GSH level (b), and lipid ROS levels (c, d) were analyzed (^∗^*P* < 0.05, ^∗∗^*P* < 0.01, and ^∗∗∗^*P* < 0.001).

To investigate the synergistic effects of erastin and vitamin C on cell proliferation, a CCK-8 assay was performed. As expected, treatment with either erastin or vitamin C moderately inhibited cell viability, while combined treatment with erastin and vitamin C significantly reduced PC cell proliferation, suggesting that the combination treatment could greatly disrupt PC growth (Figure 3(a)). Under microscopy, the synergistic effect of cytotoxicity was also confirmed by the morphological changes in PaTu8988 and BxPC3 cells (Figures S3A and B). GSH depletion leads to the accumulation of ROS, which triggers lipid peroxidation to generate end products such as MDA and consequently ferroptosis. To further investigate the synergetic effect of erastin and vitamin C-induced ferroptosis, the levels of GSH, MDA, and lipid ROS production were measured. The GSH levels were decreased in PaTu8988 and BxPC3 cells treated with erastin or vitamin C for 24 h, and this reduction was exacerbated by combination treatment with erastin and vitamin C (Figure 3(b)). In addition, erastin or vitamin C could lead to MDA and lipid ROS accumulation in PaTu8988 and BxPC3 cells compared with the control group, while the generation of MDA and lipid ROS was obviously increased by cotreatment with erastin and vitamin C (Figures 3(c)C and 3(d) and S3C–D).

### 3.4. Vitamin C Inhibits Erastin-Induced Ferroptosis in Normal Pancreatic Cells and Mouse Embryonic Fibroblasts

**Figure 4:**
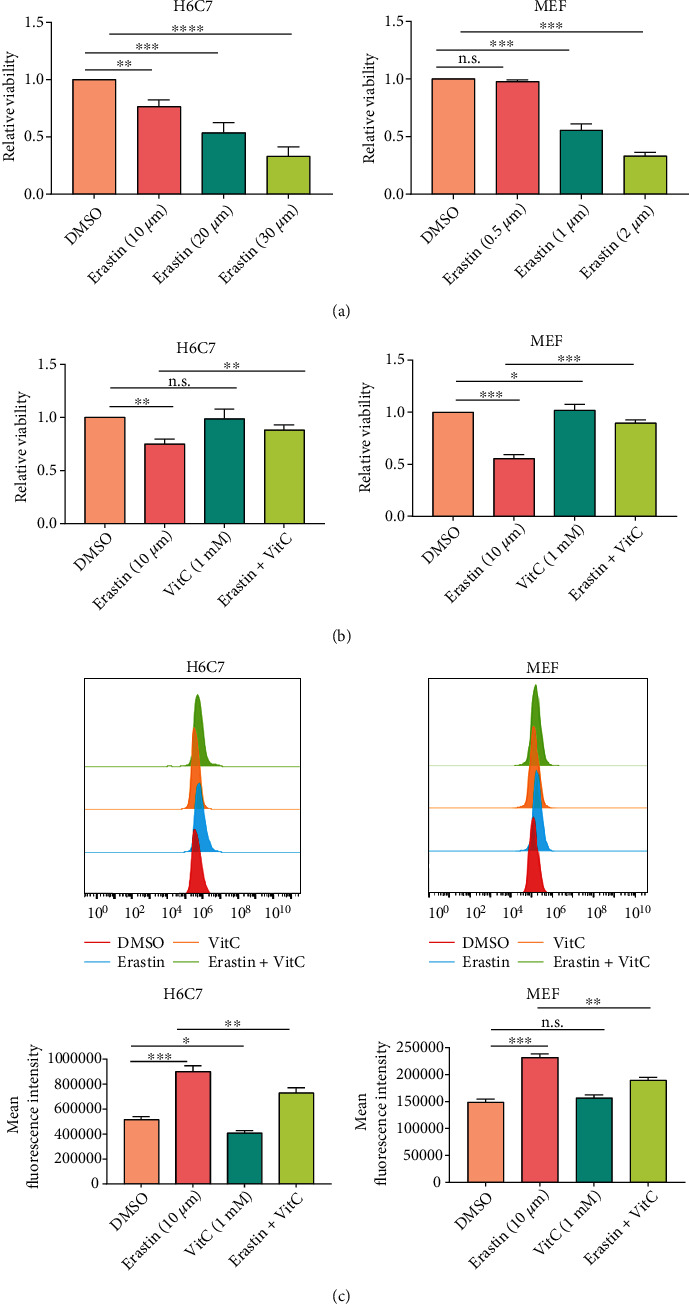
Vitamin C protected MEFs and H6C7 cells from erastin-induced ferroptosis. (a) Cell viability was assessed by CCK-8 assay after treatment with various concentrations of erastin. (b, c) H6C7 and MEF cells were treated with erastin, vitamin C, or a combination of both for 24 h. Cell viability (b) and lipid ROS levels (c) were analyzed (^∗^*P* < 0.05, ^∗∗^*P* < 0.01, and ^∗∗∗^*P* < 0.001).

Given that erastin could trigger ferroptosis in murine pancreatic *β* cells and worsen insulin secretion by pancreatic *β* cells in type 2 diabetes [30, 31], we then investigated whether erastin is cytotoxic to normal human pancreatic cells. We treated H6C7 cells with 0, 10, 20, or 30 *μ*M erastin for 24 h, and we also repeated this experiment in MEFs. The CCK-8 assay showed that erastin inhibited the growth of H6C7 cells and MEFs in a dose-dependent manner, suggesting that high-dose erastin may be cytotoxic to normal cells (Figure 4(a)). A significant amount of clinical data consistently show that vitamin C treatment could simultaneously act as a sensitizing agent of cancer cells to chemoradiation and function as a protectant of normal tissues from chemoradiation [32]. Given the synergistic effect of erastin with vitamin C-induced ferroptosis, we speculated whether vitamin C combined with erastin would protect normal cells from ferroptosis. To verify our hypothesis, we first performed CCK-8 assays to analyze the proliferative capacity of H6C7 cells and MEFs. The results showed that vitamin C rescued the cell death of H6C7 and MEF cells caused by erastin (Figure 4(b)). This was further confirmed by the morphological changes in H6C7 cells and MEFs (Figures S4A and B). In addition, a higher GSH elevation and lower MDA and lipid ROS generation were observed in the cells treated with the combination of erastin and vitamin C than in the cells treated with erastin alone (Figures 4(c) and S4C–F). These results indicated that vitamin C could protect H6C7 cells and MEFs from erastin-induced ferroptosis.

### 3.5. Combined Treatment with Erastin and Vitamin C Synergistically Increases Intracellular Ferrous Iron by Regulating the AMPK/NRF2/HMOX1 Axis

**Figure 5:**
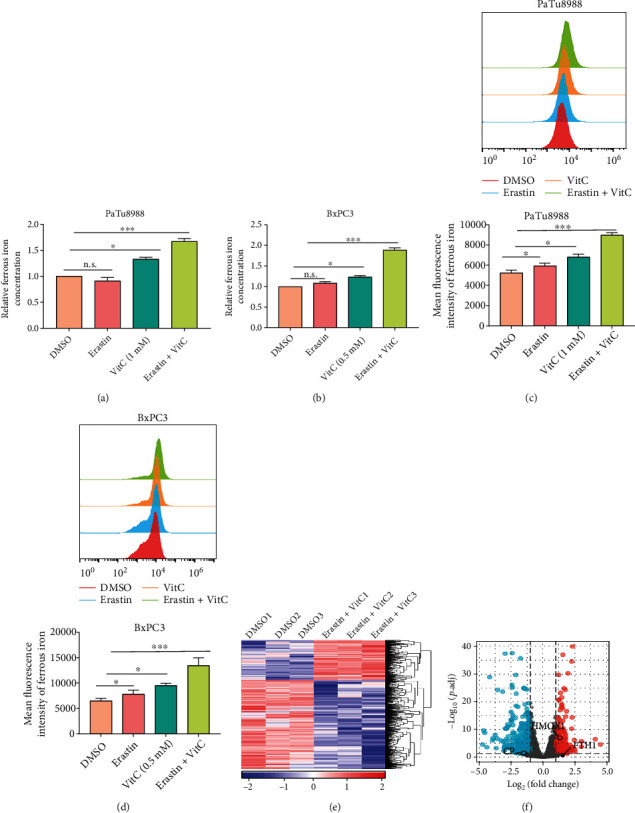
Erastin and vitamin C increase the intracellular ferrous iron level. (a, b) The intracellular ferrous iron level was detected by an iron assay kit in PaTu8988 and BxPC3 cell lines under treatment with erastin and vitamin C. (c, d) Flow cytometry was performed to measure ferrous iron levels, and the quantification of fluorescence intensity is shown (^∗^*P* < 0.05, ^∗∗∗^*P* < 0.001). (e, f) Heat map and volcano map analyses showed differentially expressed genes in response to erastin and vitamin C treatment measured by RNA-seq, respectively. Blue: low expression levels. Red: high expression levels.

However, the mechanism of the synergistic effect of erastin and vitamin C on ferroptosis induction is still unclear. As a major hallmark of ferroptosis, an accumulated labile iron pool (LIP), mainly in the form of ferrous iron (Fe^2+^), can promote lipid peroxidation and catalyze the decomposition of H_2_O_2_ to generate highly reactive hydroxyl radicals (^·^OH) through the Fenton reaction to aggravate intracellular ROS formation and induce ferroptosis. Indeed, vitamin C can function as a prooxidant agent in the presence of Fe^2+^, and it can also provide electrons to ferric iron (Fe^3+^, oxidized) to regenerate Fe^2+^ (reduced), resulting in a positive feedback loop to contribute to cell death. To examine whether the synergistic effect of erastin and vitamin C is due to the increase in LIP, we assessed the intracellular ferrous iron level in PC cells treated with erastin and vitamin C. Compared with erastin or vitamin C treatment alone, LIP levels were apparently elevated in Patu8988 and BxPC3 cell lines under treatment with erastin and vitamin C (Figures 5(a) and 5(b)). The selective fluorescence imaging probe BioTracker 575 Red Fe^2+^ Dye further confirmed that erastin combined with vitamin C significantly upregulated LIP accumulation in PC cells (Figures 5(c) and 5(d)). To further explore the potential molecular mechanisms, RNA sequencing (RNA-seq) analysis was performed to analyze the gene expression changes in BxPC3 cells treated with erastin and vitamin C. The heat map analysis of differentially expressed genes revealed that 288 genes were upregulated and 644 genes were downregulated in the erastin and vitamin C treatment groups compared with the DMSO treatment group (Figure 5(e)). Expanded lists of differentially expressed genes are provided in Supplementary Table S3. We focused on the genes related to iron metabolism for downstream studies, including HMOX1, CP, and FTH1 (Figure 5(f)).

**Figure 6:**
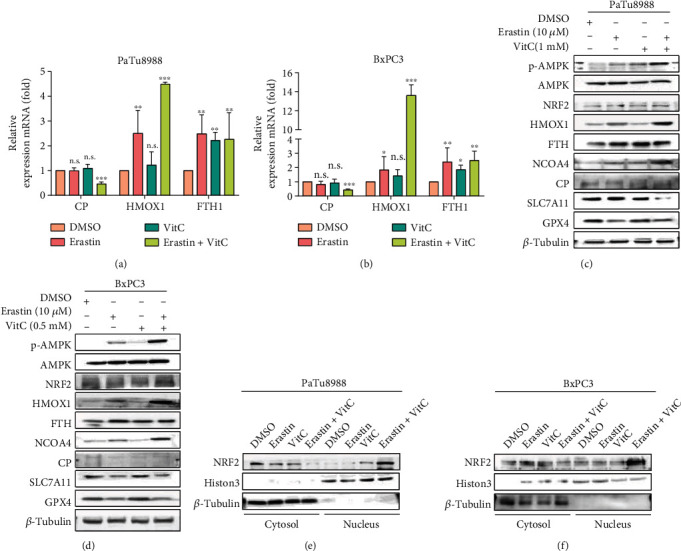
Erastin and vitamin C trigger ferroptosis by regulating the AMPK/NRF2/HMOX1 pathway. (a, b) The mRNA levels of CP, HMOX1, and FTH1 were analyzed in Patu8988 and BxPC3 cell lines treated with erastin and/or vitamin C. (c, d) Ferroptosis and iron metabolism-related proteins were detected by Western blot analysis. (e, f) Western blotting analysis of the nuclear/cytoplasmic fraction showed NRF2 nuclear localization in Patu8988 and BxPC3 cells after treatment with erastin and/or vitamin C (^∗^*P* < 0.05, ^∗∗^*P* < 0.01, and ^∗∗∗^*P* < 0.001).

To verify the RNA-sequencing data, we determined the expression of these genes by qPCR and Western blotting. Consistent with the results of RNA sequencing, the mRNA levels of HMOX1 and FTH1 were significantly increased, while CP expression was decreased in PaTu8988 and BxPC3 cells after cotreatment with erastin and vitamin C (Figures 6(a) and 6(b)). Western blotting showed that erastin and vitamin C cotreatment increased HMOX1, FTH1, NCOA4, and phospho-AMPK levels without affecting CP in PaTu8988 and BxPC3 cells, as shown in Figures 6(c) and 6(d). NCOA4, which is required for the delivery of ferritin into the lysosome, leads to iron release [33]. This might be the reason why FTH1 expression in the cotreatment group did not show a significant difference from that in the erastin- or vitamin C-treated group. Elevated ROS levels result in the activation of AMPK and can facilitate the nuclear accumulation of NRF2 [34, 35]. As a transcription factor, NRF2 nuclear translocation transactivates its target genes, especially HMOX1. To analyze the cellular location of NRF2, we performed nuclear/cytoplasmic extraction and immunofluorescence. The results showed that NRF2 translocation into the nucleus was observed in the erastin and vitamin C combination-treated group (Figures 6(e) and 6(f) and S5A–B). These results suggested that the AMPK/NRF2/HMOX1 signaling pathway was activated with cotreatment with erastin and vitamin C.

### 3.6. Erastin and Vitamin C Treatment Inhibited Tumor Growth In Vivo

**Figure 7:**
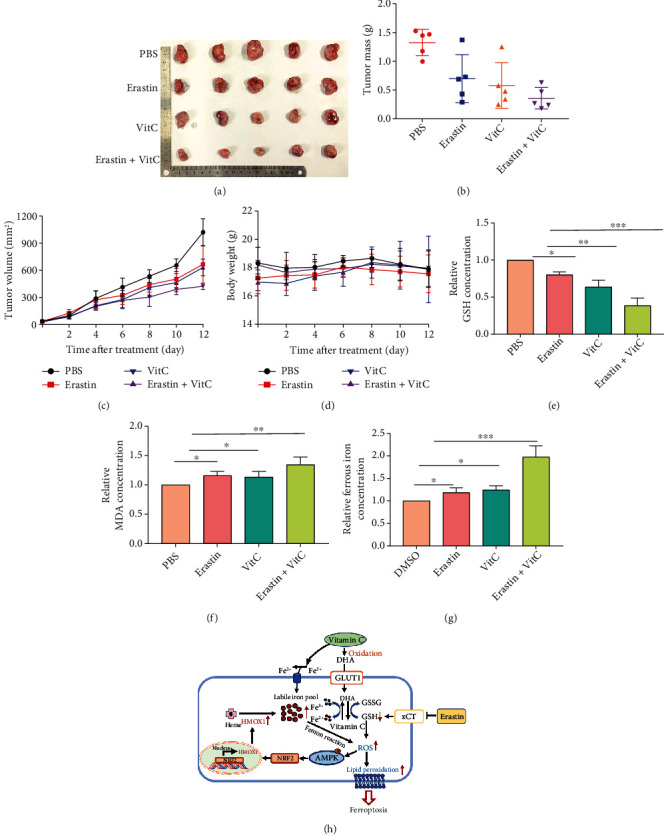
Vitamin C enhances erastin-induced ferroptosis in pancreatic cancer cells in vivo. (a, b) Images and weights of xenograft tumors from various mouse groups after 14 days of treatment. (c, d) The tumor volume and mouse body weight were measured every two days. (e–g) Combination treatment with erastin and vitamin C reduced GSH (e) and increased the MDA (e) and ferrous iron levels (g). (h) A schematic showing that vitamin C sensitizes PC cells to erastin-induced ferroptosis via GSH depletion and ferrous iron overload (^∗^*P* < 0.05, ^∗∗^*P* < 0.01, and ^∗∗∗^*P* < 0.001).

In addition to the direct antitumor effect of vitamin C at high doses, recent studies have shown that the effect of vitamin C on immune cells can mediate indirect antitumor effects [24, 36]. To determine the lethal effect of erastin and vitamin C on PC in vivo, we implanted Panc02 cells into C57BL/6 mice (5 mice in each group) followed by the cotreatment of erastin and/or vitamin C. We also treated Panc02 cells with different concentrations of erastin or vitamin C for 24 h and assessed cell viability via a CCK-8 assay. The results indicated that both erastin and vitamin C induced cell death in a dose-dependent manner in Panc02 cells (Figures S6A–B). In addition, combined treatment with erastin and vitamin C significantly reduced Panc02 cell proliferation (Figure S6C). Tumor growth was monitored from days 1 to 15 after treatment, and the results demonstrated that the xenograft tumors in the erastin and vitamin C combination-treated group were much smaller than those in either the erastin or vitamin C monotreated group (Figures 7(a) and 7(b)). Erastin and vitamin C treatment significantly suppressed tumor growth (Figure 7(c)). Among the groups, there was an insignificant difference in body weight (Figure 7(d)). In addition, erastin and vitamin C treatment also caused reduced the GSH level and increased the MDA and ferrous iron levels (Figures 7(e)–7(g)). H&E staining demonstrated that erastin and vitamin C did not damage the heart, liver, spleen, lungs, or kidneys at the given dose (Figure S7).

## 4. Discussion

The present study demonstrates that pharmacologic concentrations of vitamin C can selectively induce ferroptosis in PC cells but not normal pancreatic ductal epithelial cells or mouse embryonic fibroblasts. Importantly, high-dose vitamin C combined with erastin can synergistically suppress the proliferation of PC cells. At the same time, it inhibits the cytotoxicity of erastin to normal pancreatic ductal epithelial cells and mouse embryonic fibroblasts. DHA, the oxidized form of vitamin C, can be transported into PC cells expressing high levels of GLUT1, resulting in cell death. Furthermore, cotreatment with erastin and vitamin C upregulates HMOX1 expression through the AMPK/NRF2 pathway to increase the ferrous iron level. These results suggest that vitamin C can sensitize erastin-induced ferroptosis via GSH depletion and ferrous iron accumulation to exert antitumor effects in PC (Figure 7(h)).

Ferroptosis is a newly discovered type of regulated cell death that occurs in an iron-dependent manner and is characterized by the Fenton reaction catalyzed by LIP, increased ROS production, and lipid peroxidation [37, 38]. It can be triggered by various ferroptosis inducers, including erastin [13]. Unlike other ferroptosis inducers, erastin can trigger multiple molecules, including system Xc^−^, VDAC, and p53, to kill cancer cells efficiently, suggesting a promising future in tumor therapeutic strategies [39]. Due to irregular iron metabolism, lipid metabolism, and altered antioxidant defenses, different patterns of ferroptosis sensitivity have been observed in various types of cancer cells [40–42]. Previous studies have demonstrated that a variety of cancer cells are insensitive to erastin-induced ferroptosis. Our study also found that the PC cell lines PaTu8988 and BxpC3 are insensitive to erastin, and normal pancreatic cells can be affected by high-dose erastin. Therefore, it is imperative to develop novel therapeutics with more effective and less toxic therapies to sensitize cancer cells to erastin.

Numerous studies and clinical trials have demonstrated the safety and efficacy of pharmacological vitamin C as monotherapy or component of combination therapy in anticancer treatment over the past 50 years [43]. Cameron and Pauling found that vitamin C could protect against the spread of cancer cells by promoting the production of serum physiological hyaluronidase inhibitors in the early 1970s [44]. A high dose of vitamin C can act as a prooxidant to exert antitumor effects, and it is oxidized to DHA in the extracellular microenvironment. DHA is transported into the cell via GLUTs, and it can be rapidly reduced to vitamin C by decreasing GSH activity and NADPH and generating ROS [45]. This antitumour mechanism reminds us of ferroptosis, and we hypothesized that high-dose vitamin C could trigger ferroptosis in PC cells. In the present study, a high dose of vitamin C was observed to effectively suppress the proliferation of PC cells but not normal pancreatic ductal epithelial cells or mouse embryonic fibroblasts. Further experiments revealed that vitamin C could induce ferroptosis by GSH depletion and ROS generation in PC cells.

Some cancer cells are more sensitive to vitamin C, and this sensitivity is characterized by increased levels of labile ferrous iron, glycolysis addiction, and GLUT1 upregulation. To examine how vitamin C selectively induces cell death in PC cells, we first analyzed the expression of the main transporter of DHA-GLUT1 in PC tissues and cell lines. We found that GLUT1 is highly expressed in human PC tissues and cell lines, and its expression is positively correlated with poorer patient outcomes. GLUT1 knockdown can partly prevent cell death induced by vitamin C, suggesting that GLUT1 plays an essential role in vitamin C selectively inducing cell death in PC cells.

Given that vitamin C can sensitize cancer cells to tumor therapy and protect normal tissue, we investigated whether vitamin C combined with erastin synergistically induced PC cell death while limiting side effects. Indeed, the present study demonstrated that vitamin C acts synergistically with erastin to suppress the proliferation of PaTu8988 and BxPC3 cells accompanied by GSH depletion, excessive ROS generation, and lipid peroxide accumulation. In addition, vitamin C protects against erastin-induced ferroptosis in H6C7 cells and MEFs.

As an important factor in the formation of ROS, the iron redox cycle between ferrous iron (Fe^2+^, reduced) and ferric iron (Fe^3+^, oxidized) plays a vital role in the sensitivity of cells to ferroptosis. Excessive ferrous iron can directly catalyze the decomposition of H_2_O_2_ to generate hydroxyl radicals (^·^OH) through the Fenton reaction while accelerating ROS production and inducing ferroptosis. Vitamin C can effectively provide electrons to Fe^3+^ to regenerate Fe^2+^ to perpetuate this reaction, and a high dose of vitamin C can be fully oxidized to DHA in the presence of Fe^2+^, which generates extracellular H_2_O_2_ and forms a positive feedback loop to contribute to cell death. Additionally, extracellular H_2_O_2_ can diffuse into cancer cells and react with intracellular LIP to generate ^·^OH. In addition, DHA is transported into cancer cells via high expression of GLUT1 due to its structural similarity to glucose, and it can efficiently reduce vitamin C consuming GSH and NADPH, resulting in excess ROS production. Due to altered iron metabolism, cancer cells enriched with high labile iron levels (such as pancreatic cancer and breast cancer) are more sensitive to ferroptosis inducers [46, 47]. The results from our study suggested that erastin combined with vitamin C significantly depleted GSH and accelerated the accumulation of LIP in PaTu8988 and BxPC3 cells.

Many aspects of iron metabolism, including iron uptake, storage, utilization, and efflux, strictly control the sensitivity of ferroptosis in cancer cells. Thus, we hypothesized that regulators of iron metabolism might be altered after erastin and vitamin C treatment in PC cells. The RNA-seq analysis data in this study verified our hypothesis. We found that HMOX1 is upregulated in BxPC3 cells treated with erastin and vitamin C. Heme oxygenase-1 (HMOX1) degrades heme to generate biliverdin, carbon monoxide (CO), and ferrous iron. Erastin can induce HMOX1 expression, and HMOX1 overexpression also accelerates erastin-induced ferroptosis by increasing ferrous iron levels and lipid peroxidation [48, 49]. As the most important activator of HMOX1, nuclear factor erythropoietin-2-related factor 2 (NRF2) can also be involved in iron metabolism by regulating FTH1 and FPN [50, 51]. Activation of AMPK can mediate the nuclear accumulation of NRF2 and exert a positive influence on the NRF2/HMOX1 pathway [52]. We found that oxidative stress induced by erastin and vitamin C can enhance AMPK phosphorylation, NRF2 nuclear translocation, and HMOX1 expression to increase the labile iron level, while iron addition accelerates ferroptosis through the Fenton reaction. This result indicated that the combination of vitamin C and erastin might enhance ferroptosis via the AMPK/NRF2/HMOX1 pathway. FTH1 is the major intracellular iron storage protein that can convert Fe^2+^ to Fe^3+^, and nuclear receptor coactivator 4 (NCOA4) can recruit FTH1 for autophagic degradation and iron release, contributing to ferroptosis [53]. Our study revealed that FTH1 was significantly higher in the erastin-treated and cotreatment groups than in the control group, whereas FTH1 expression in the cotreatment group did not show a significant difference from that in the monotreated groups. However, we observed that the protein level of NCOA4 was upregulated in the erastin and vitamin C combination-treated group compared with the erastin or vitamin C monotreated group, which can deliver FTH1 into the lysosome to increase labile ferrous iron. This may explain why FTH1 expression in the erastin- and vitamin C-treated groups was not significantly different from that in the erastin- or vitamin C-treated group. Our study provides evidence that the combination of erastin and vitamin C mainly increases the levels of ferrous iron through the AMPK/NRF2/HMOX1 signaling pathway. Cotreatment with erastin and vitamin C also exhibited a synergistic effect in a pancreatic cancer xenograft model in mice.

Taken together, the data from the present study demonstrated that vitamin C could intensively sensitize erastin-induced ferroptosis in PC cells and significantly reduce its cytotoxicity in normal cells. In particular, combinatory treatment with erastin and vitamin C synergistically induced ferroptosis involving GSH depletion and accumulated ferrous iron. In short, cotreatment with erastin and vitamin C provides a novel therapeutic strategy for pancreatic cancer.

## Data Availability

The RNA-seq data were deposited into Gene Expression Omnibus (GEO) database with accession number GSE192362.

## Supplementary Materials

Supplementary 1 Figure S1: (A) cell viability was assessed by CCK-8 assay after treatment with various concentrations of erastin. (B) Cell viability was detected in PaTu8988 cells after treatment with vitamin C with or without different cell death inhibitors. (C) GSH levels were measured in BxPC3 cells treated with vitamin C in the presence or absence of DFO. (D) Flow cytometry was performed to detect lipid ROS levels, and the quantification of fluorescence intensity is shown (^∗∗^*P* < 0.01, ^∗∗∗^*P* < 0.001). Figure S2: (A–D) GLUT1 mRNA and protein levels were reduced in PaTu8988 and BxPC3 cells after sh-GLUT1 transfection. (E, F) The level of GLUT1 was increased in H6C7 and PANC1 cells after GLUT1 overexpression. (G, H) Cell viability was detected in GLUT1-upregulated H6C7 and PANC1 cells treated with or without vitamin C (^∗^*P* < 0.05, ^∗∗^*P* < 0.01, and ^∗∗∗^*P* < 0.001). Figure S3: (A, B) morphological changes in PaTu8988 and BxPC3 cells after erastin/vitamin C treatment. (C, D) MDA levels were measured in PaTu8988 and BxPC3 cells under mono- or combination treatment with erastin and vitamin C. (^∗^*P* < 0.05, ^∗∗^*P* < 0.01, and ^∗∗∗^*P* < 0.001). Figure S4: (A, B) morphological changes in H6C7 cells and MEFs after treatment with erastin and/or vitamin C. (C–F) GSH and MDA levels were assayed in H6C7 cells and MEFs under mono- or combination treatment with erastin and vitamin C (^∗^*P* < 0.05, ^∗∗^*P* < 0.01, and ^∗∗∗^*P* < 0.001). Figure S5: (A, B) immunofluorescence revealed the subcellular localization of NRF2 in PaTu8988 and BxPC3 cells treated with erastin and/or vitamin C. Figure S6: (A, B) cell viability was assessed by CCK-8 assay after treatment with various concentrations of vitamin C or erastin. (C) Cell viability was assessed by CCK-8 assay after treatment with erastin, vitamin C, or a combination of both in Panc02 cells. Figure S7: H&E staining of the hearts, liver, spleens, lungs, and kidneys.

Supplementary 2 Table S1: shRNAs used in this study.

Supplementary 3 Table S2: primer sequences used for qRT-PCR.

Supplementary 4 Table S3: lists of differentially expressed genes.
