# Supplementary material for: Vitamin C Sensitizes Pancreatic Cancer Cells to Erastin-Induced Ferroptosis by Activating the AMPK/Nrf2/HMOX1 Pathway
Source: Oxid Med Cell Longev. 2022 Jul 19;2022:5361241. doi: 10.1155/2022/5361241 (PMC9338737; doi:10.1155/2022/5361241)

**Supporting information**

Figure S1.

(A) Cell viability was assessed by CCK-8 assay after treatment with various concentrations of erastin. (B) Cell viability was detected in PaTu8988 cells after treatment with vitamin C with or without different cell death inhibitors. (C) GSH levels were measured in BxPC3 cells treated with vitamin C in the presence or absence of DFO. (D) Flow cytometry was performed to detect lipid ROS levels, and the quantification of fluorescence intensity is shown. (***P*<0.01, *** *P*<0.001).

Figure S2.

(A-D) GLUT1 mRNA and protein levels were reduced in PaTu8988 and BxPC3 cells after sh-GLUT1 transfection. (E-F) The level of GLUT1 was increased in H6C7 and PANC1 cells after GLUT1 overexpression. (G-H) Cell viability was detected in GLUT1-upregulated H6C7 and PANC1 cells treated with or without vitamin C. (**P*<0.05, ***P*<0.01, *** *P*<0.001).

Figure S3.

(A-B) Morphological changes in PaTu8988 and BxPC3 cells after erastin/vitamin C treatment. (C-D) MDA levels were measured in PaTu8988 and BxPC3 cells under mono or combination treatment with erastin and vitamin C. (**P*<0.05, ***P*<0.01, *** *P*<0.001).

Figure S4.

(A-B) Morphological changes in H6C7 cells and MEFs after treatment with erastin and/or vitamin C. (C-F) GSH and MDA levels were assayed in H6C7 cells and MEFs under mono or combination treatment with erastin and vitamin C. (**P*<0.05, ***P*<0.01, *** *P*<0.001).

Figure S5.

(A-B) Immunofluorescence revealed the subcellular localization of NRF2 in PaTu8988 and BxPC3 cells treated with erastin and/or vitamin C.

Figure S6.

(A-B) Cell viability was assessed by CCK-8 assay after treatment with various concentrations of vitamin C or erastin. (C) Cell viability was assessed by CCK-8 assay after treatment with erastin, vitamin C, or a combination of both in Panc02 cells.

Figure S7.

H&E staining of hearts, liver, spleens, lungs, and kidneys.


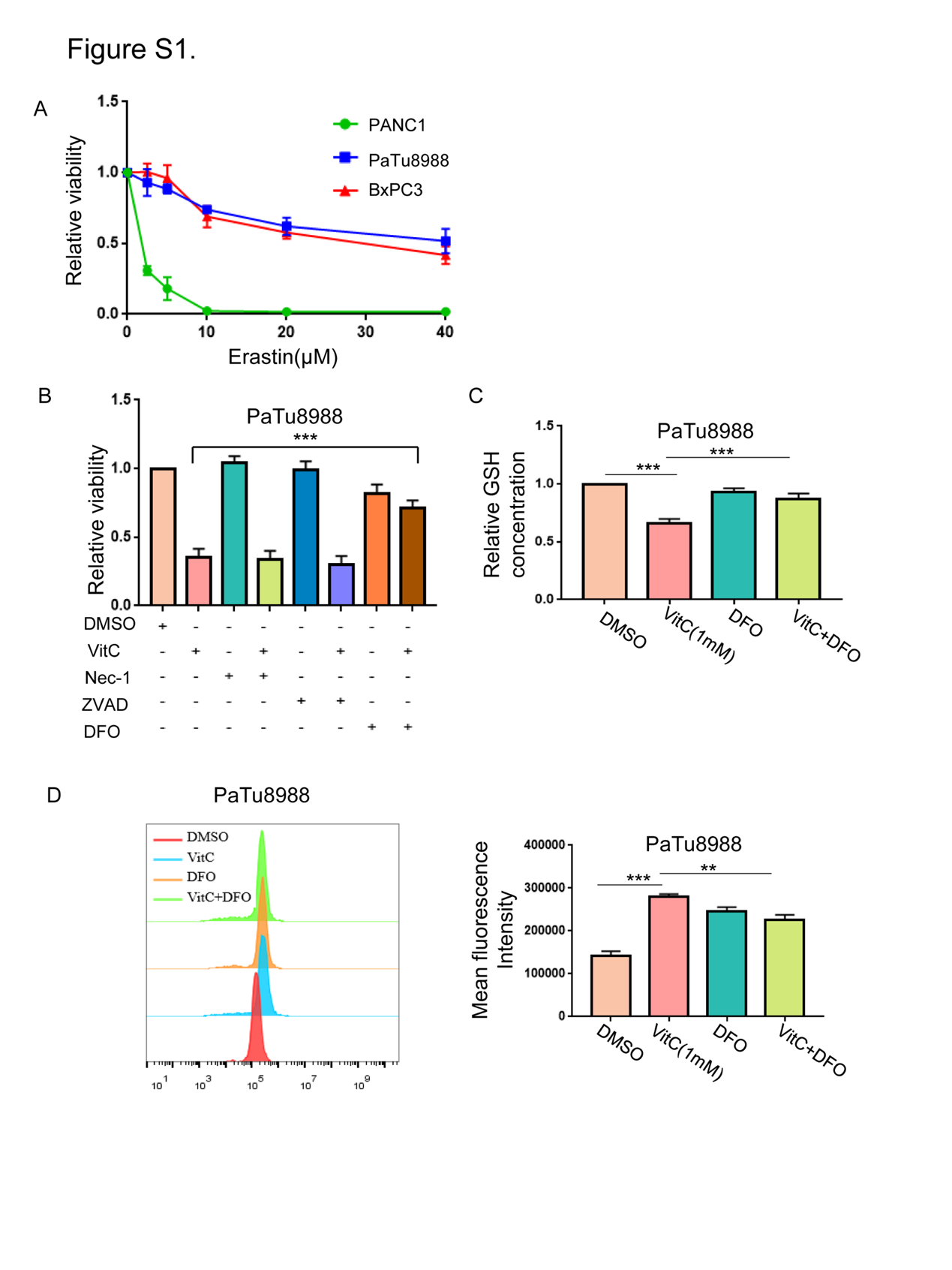


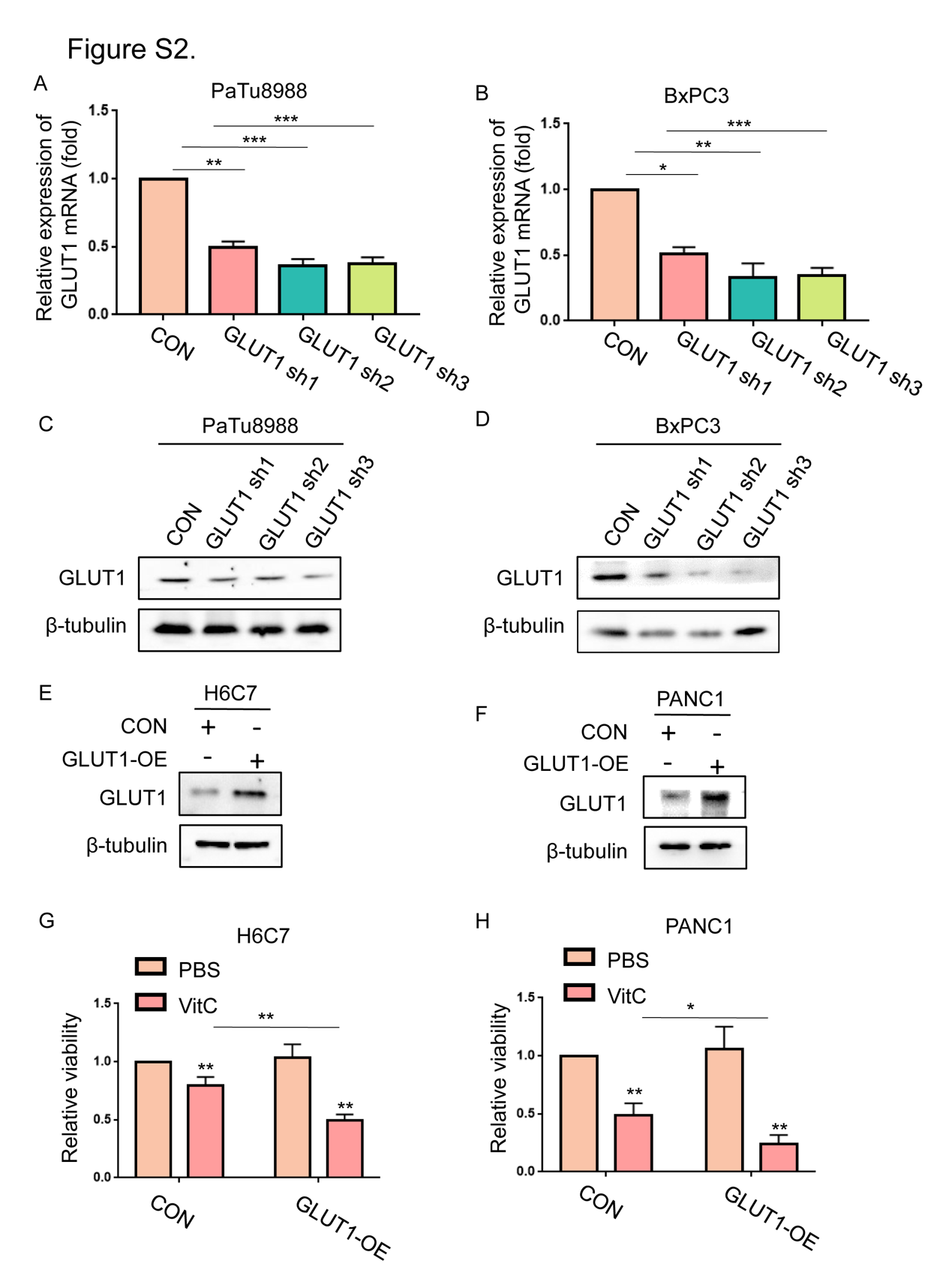


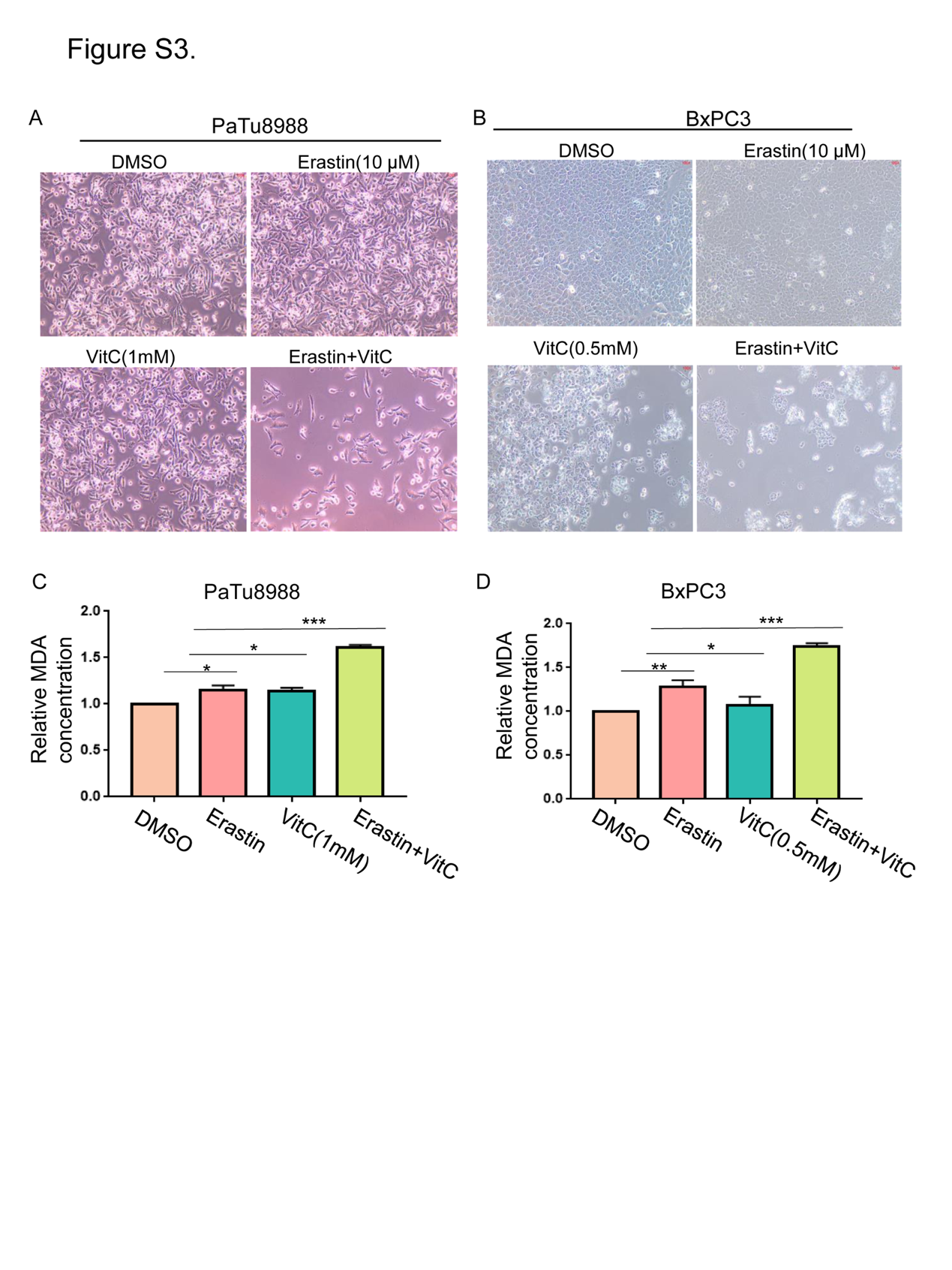


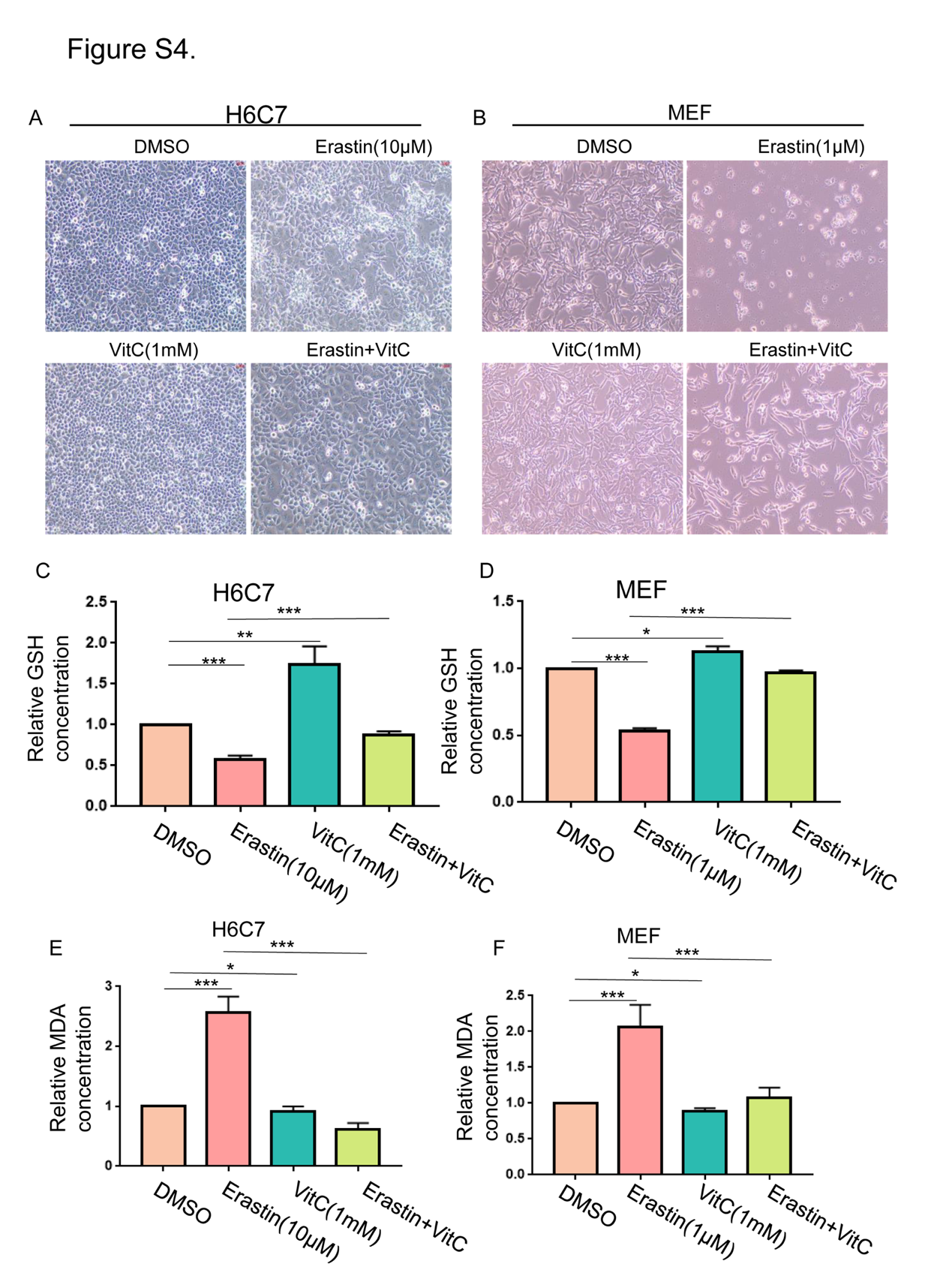


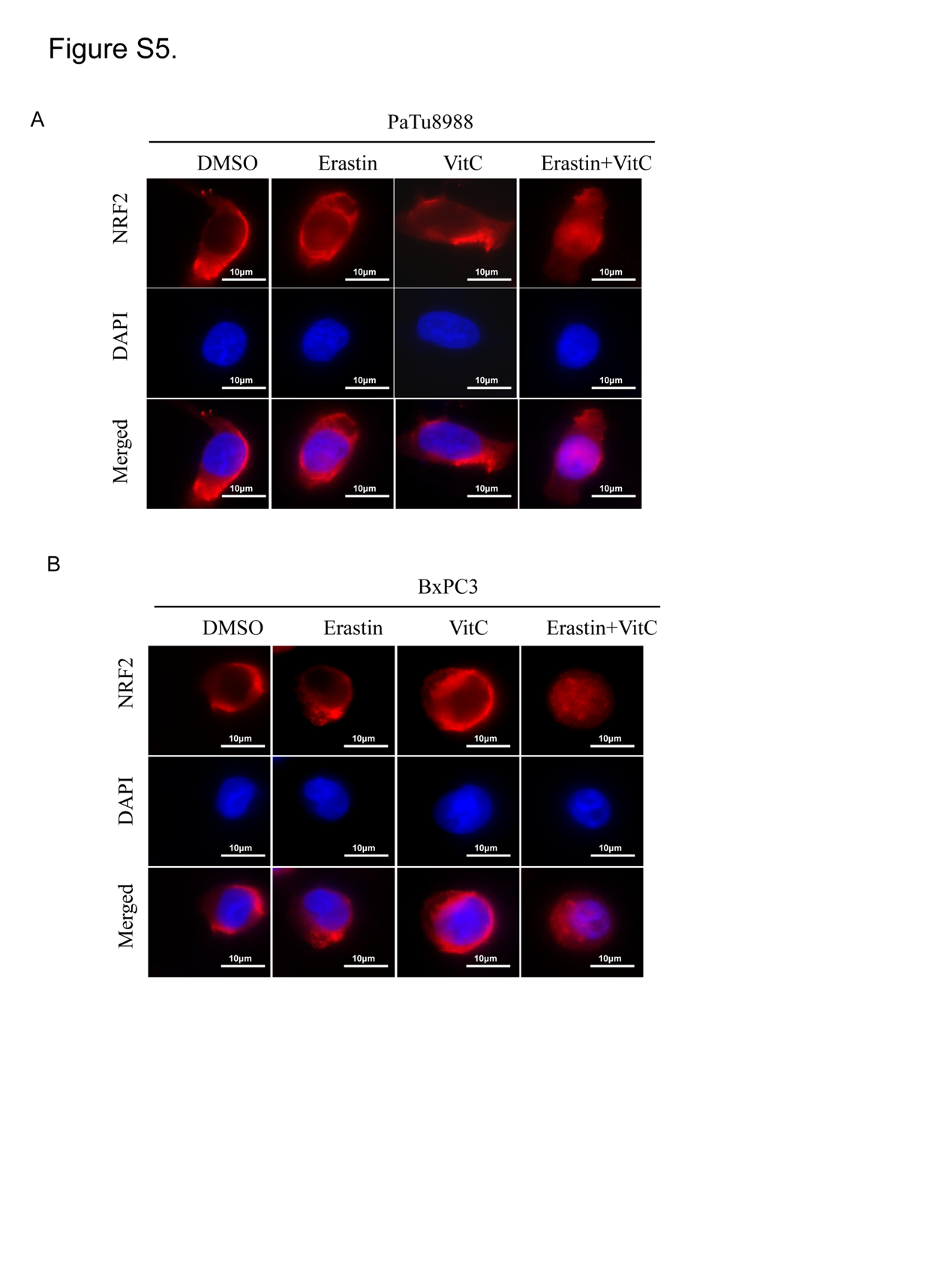


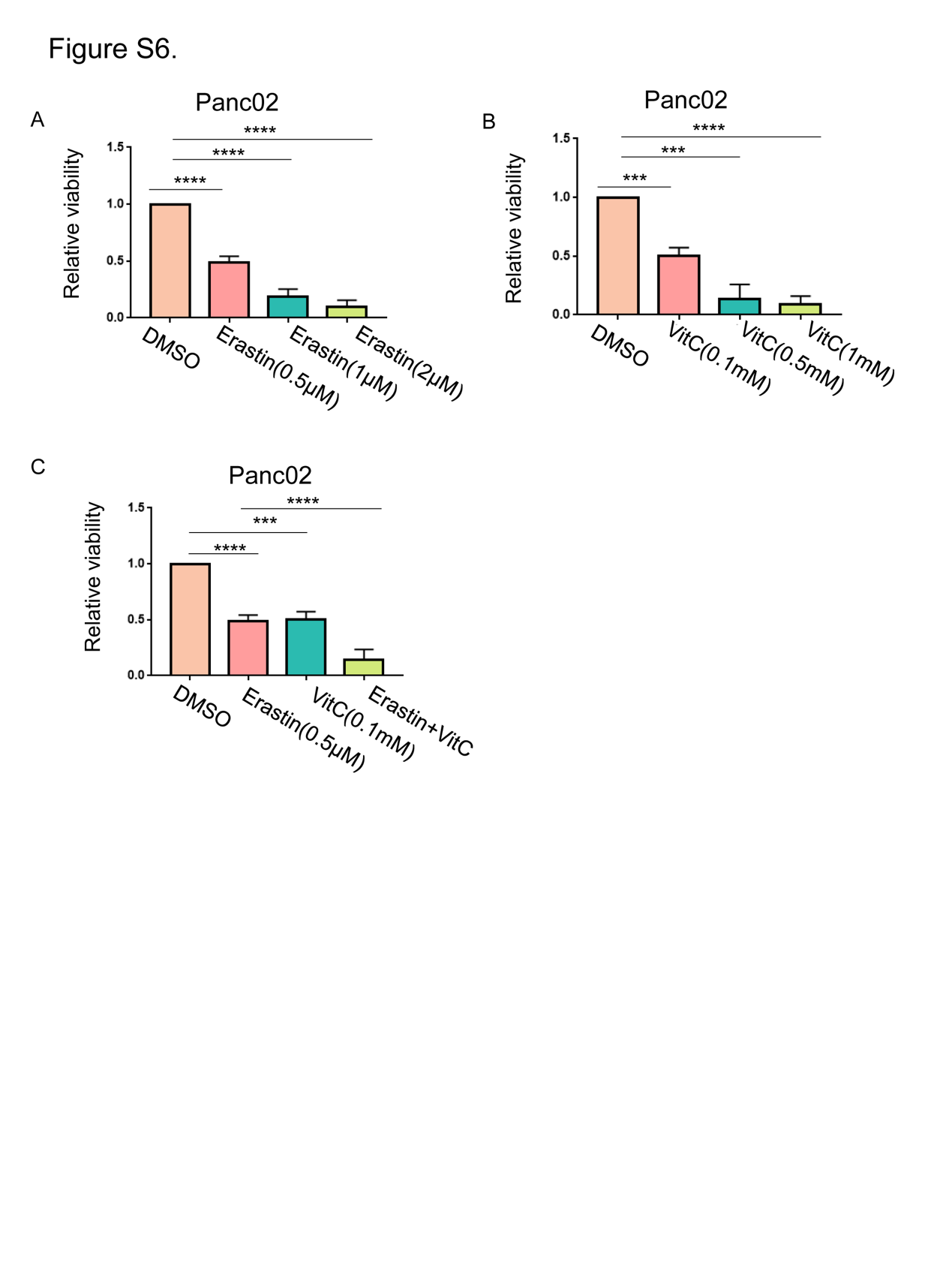


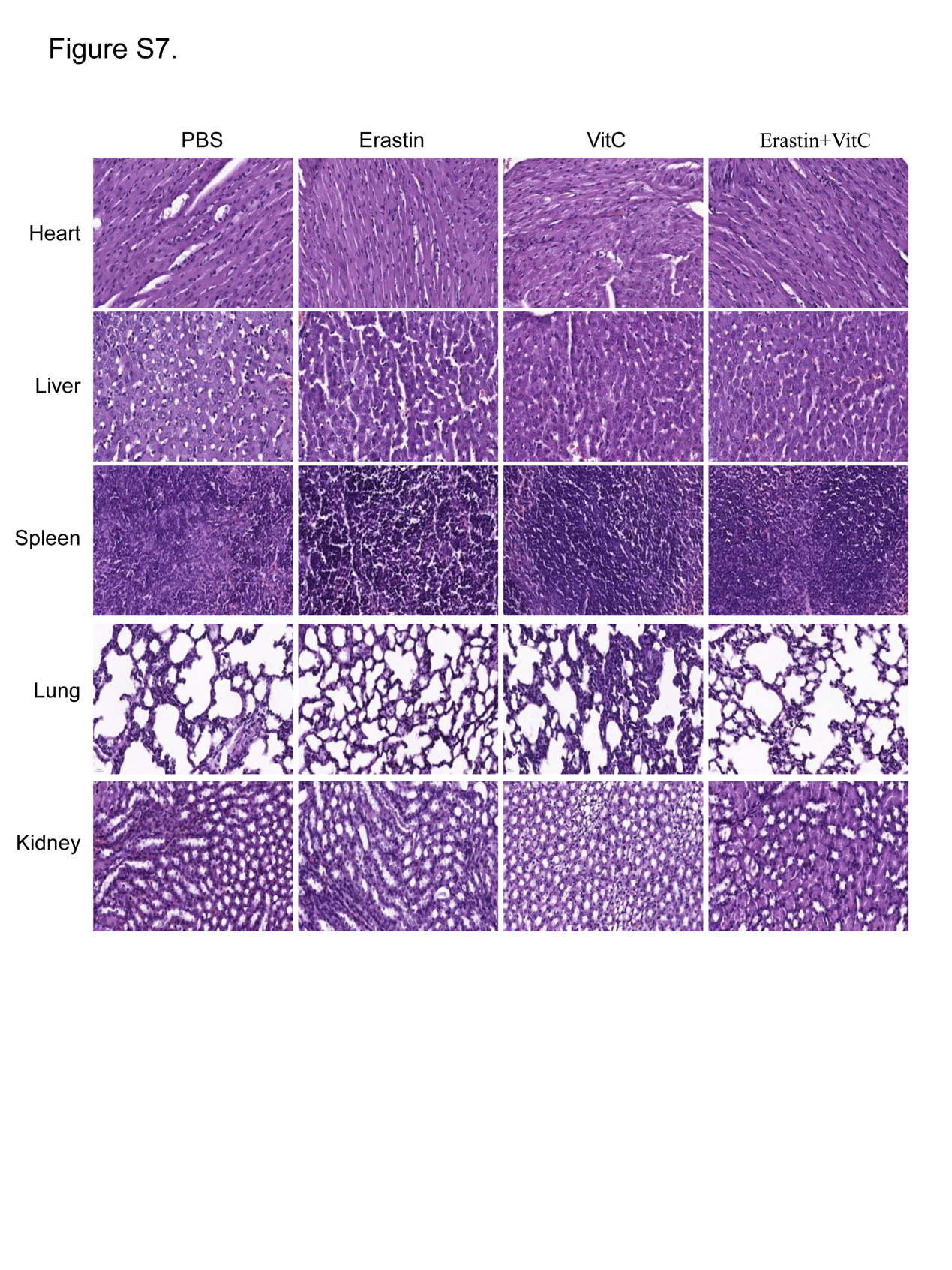

Supplement: Supplementary 1 — Figure S1: (A) cell viability was assessed by CCK-8 assay after treatment with various concentrations of erastin. (B) Cell viability was detected in PaTu8988 cells after treatment with vitamin C with or without different cell death inhibitors. (C) GSH levels were measured in BxPC3 cells treated with vitamin C in the presence or absence of DFO. (D) Flow cytometry was performed to detect lipid ROS levels, and the quantification of fluorescence intensity is shown (∗∗P < 0.01, ∗∗∗P < 0.001). Figure S2: (A–D) GLUT1 mRNA and protein levels were reduced in PaTu8988 and BxPC3 cells after sh-GLUT1 transfection. (E, F) The level of GLUT1 was increased in H6C7 and PANC1 cells after GLUT1 overexpression. (G, H) Cell viability was detected in GLUT1-upregulated H6C7 and PANC1 cells treated with or without vitamin C (∗P < 0.05, ∗∗P < 0.01, and ∗∗∗P < 0.001). Figure S3: (A, B) morphological changes in PaTu8988 and BxPC3 cells after erastin/vitamin C treatment. (C, D) MDA levels were measured in PaTu8988 and BxPC3 cells under mono- or combination treatment with erastin and vitamin C. (∗P < 0.05, ∗∗P < 0.01, and ∗∗∗P < 0.001). Figure S4: (A, B) morphological changes in H6C7 cells and MEFs after treatment with erastin and/or vitamin C. (C–F) GSH and MDA levels were assayed in H6C7 cells and MEFs under mono- or combination treatment with erastin and vitamin C (∗P < 0.05, ∗∗P < 0.01, and ∗∗∗P < 0.001). Figure S5: (A, B) immunofluorescence revealed the subcellular localization of NRF2 in PaTu8988 and BxPC3 cells treated with erastin and/or vitamin C. Figure S6: (A, B) cell viability was assessed by CCK-8 assay after treatment with various concentrations of vitamin C or erastin. (C) Cell viability was assessed by CCK-8 assay after treatment with erastin, vitamin C, or a combination of both in Panc02 cells. Figure S7: H&E staining of the hearts, liver, spleens, lungs, and kidneys. [file 5361241.f1.docx]
